# A Prospective Evaluation of Quick Intraoperative Parathyroid Hormone Assay at the Time of Skin Closure in Predicting Clinically Relevant Hypocalcemia after Thyroidectomy

**DOI:** 10.1007/s00268-012-1561-9

**Published:** 2012-03-08

**Authors:** Brian Hung-Hin Lang, Patricia Chun-Ling Yih, Ka Kin Ng

**Affiliations:** 1 Division of Endocrine Surgery, Department of Surgery, University of Hong Kong, Pokfulam, Hong Kong SAR, China; 2 Division of Endocrine Surgery, Department of Surgery, Queen Mary Hospital, 102 Pokfulam Road, Hong Kong SAR, China

## Abstract

**Background:**

Post-thyroidectomy hypocalcemia is a major contributing factor in delayed hospital discharge and dissuading surgeons from ambulatory thyroidectomy. We prospectively evaluated the accuracy and reliability of quick parathyroid hormone level measurement at skin closure (PTH-SC) in predicting clinically relevant hypocalcemia (i.e., patients requiring calcium ± calcitriol supplements on hospital discharge).

**Methods:**

Of the 117 patients who underwent a total or completion total thyroidectomy and PTH-SC, 17 (14.5 %) had hypocalcemic symptoms or adjusted calcium <1.90 mmol/L requiring calcium and/or calcitriol supplements on discharge. Serum calcium was checked regularly in the perioperative period until stabilization and an additional quick PTH was checked on the following morning (PTH-D1). Univariate and multivariate analyses were performed to evaluate potential preoperative clinicopathologic factors and postoperative day 0 biochemical indicators. Youden’s index and the area under the ROC curve (AUC) were used to determine the best cutoff value and predictability of significant variables or criteria, respectively.

**Results:**

In the multivariate analysis, low preoperative adjusted calcium (*p* = 0.041) and low PTH-SC (*p* = 0.001) were the two independent variables associated with hypocalcemia. PTH-SC (≤1 or >1 pmol/L) had a higher specificity (95.0 %) and AUC (0.887) than serial calcium monitoring or PTH-D1 alone. Although 3/98 of patients with PTH-SC >1 pmol/L required calcium supplements on discharge, they required only the minimum amount to maintain normocalcemia.

**Conclusion:**

PTH-SC is an accurate and reliable means of predicting clinically relevant hypocalcemia. It would be reasonable to discharge those with PTH-SC >1 pmol/L on the same operative day as the risk of life-threatening hypocalcemia would seem unlikely.

## Introduction

Postoperative transient hypoparathyroidism leading to hypocalcemia is the one of the most frequent morbidities following total thyroidectomy, with incidence ranging between 3 and 40 % [1, 2]. In addition, because potentially life-threatening hypocalcemia may not develop until 24–48 h after surgery, besides postoperative bleeding and hematoma formation, hypoparathyroidism is a major reason for delayed hospital discharge and dissuading surgeons from performing ambulatory thyroid surgery [3, 4]. To safely manage postoperative hypoparathyroidism/hypocalcemia, three approaches had been described, namely, serial calcium (Ca) monitoring [5, 6], routine Ca supplementation [7, 8], and parathyroid hormone (PTH)-directed supplementation [9]. Serial Ca monitoring is often adopted because Ca testing is widely available but involves patients staying for at least 1–2 nights in hospital [2, 5, 6]. In the era of cost containment, there has been a gradual shift from serial Ca monitoring to the other two approaches [7–9]. Our group previously reported that a <75 % decline in quick PTH level from preoperative to 10-min post excision could accurately identify normocalcemic patients [10]. However, this approach required the collection of several samples during surgery and it was found that the test’s accuracy varied with the preoperative PTH level [11, 12]. As a result, some authors have advocated the use of a single PTH test some time after surgery [9–13]. To date, numerous PTH criteria have been proposed but none have clearly been shown to be superior [9, 12, 13]. Recent reviews suggested that a single PTH measurement taken any time from 10 min to several hours postoperatively seemed to provide equally accurate predictive results [12, 13]. In our clinical setting, a single quick PTH level measurement taken at the time of skin closure (PTH-SC) while the patient is still anesthetized would be preferred because no extra pain is inflicted while drawing blood and the PTH result would be available sooner to facilitate ambulatory surgery. Therefore, the aim of our study was to evaluate prospectively the accuracy and reliability of quick PTH-SC in predicting clinically relevant postoperative hypocalcemia. The study first analyzed potential risk factors and biochemical indicators associated with hypocalcemia and then compared the sensitivity, specificity, and predictability of PTH-SC with other biochemical variables or criteria such as serial Ca monitoring and following-morning PTH level (PTH-D1).

## Patients and methods

This was a prospective study carried out from January to June 2011 that included all patients who underwent either a total or a completion total thyroidectomy for benign or malignant disease. All patients were operated on and cared for by the same surgical team. The present study protocol was approved by the local institutional review board. The quick PTH level measurements were taken immediately at the time of skin closure (PTH-SC) (approximately 5–10 min after thyroid gland removal) while the patient was still anesthetized, and the following morning (approximately 24 h after operation) on postoperative day 1 (PTH-D1). Serum Ca and phosphate levels were checked preoperatively, within 1 h after surgery, the following morning, and every 8–10 h until stabilization. There were 125 consecutive patients who underwent total or completion total thyroidectomy over the study period. Those with concomitant selective neck dissection (*n* = 4) and incomplete PTH values (*n* = 4) were excluded leaving 117 (93.6 %) whose data were eligible for analysis. For this study, only those with hypocalcemic symptoms and/or a serum Ca <1.90 mmol/L (normal range = 2.11–2.55 mmol/L) (i.e., clinically relevant hypocalcemia) were given 500–1,500-mg calcium tablets daily. Calcitriol 0.25 mcg twice daily was added if the 1,500-mg calcium tablets alone failed to maintain normocalcemia. To avoid treatment bias, the result of the quick PTH-SC was not made available to the person responsible for prescribing supplements. On hospital discharge, there were 100 (85.5 %) patients who did not require any oral calcium ± calcitriol (group I), while 17 (14.5 %) required some supplements (group II). To analyze possible risk factors or predictors for hypocalcemia, patients’ clinicopathological data and postoperative day 0 biochemical indicators of the patients in the two groups were compared.

Surgical techniques, postoperative care, and follow-up protocol had been previously described [2, 14]. Thyroidectomy was performed in a standardized manner under general anesthesia. The strap muscles were separated in the midline and retracted laterally. Both recurrent laryngeal nerves and parathyroid glands were routinely identified and preserved. Any devascularized parathyroid glands were immediately minced and autoimplanted to the ipsilateral sternocleidomastoid muscle. A wound drain was not used. Operative findings such as the weight of the excised thyroid gland and the number of parathyroid glands identified and autotransplanted were recorded. Oral thyroxin supplement was given on discharge. All postsurgical patients were followed up within 1 week and were asked specifically about hypocalcemic symptoms after hospital discharge. They were then followed up every 2–3 months for the first year. Those taking calcium ± calcitriol supplements were followed more frequently with the aim of slowly weaning the patient off these supplements while maintaining normocalcemia. By definition, those who discontinued all supplements in the presence of normocalcemia within 6 months of surgery were regarded as having temporary hypoparathyroidism, whereas those who continued on the supplements for more than 6 months were categorized as having permanent hypoparathyroidism.

### Laboratory methods

Serum albumin-adjusted Ca and phosphate levels were measured in the hospital laboratory by standard methods using the Roche Diagnostics Modular Analytic System (Roche Diagnostics, Indianapolis, IN, USA). Quick PTH level was measured by the Access^®^ 2 immunoassay system (Beckman Coulter, Brea, CA, USA), and the inter- and intra-assay CVs were 5.8 and 4.5 %, respectively. The normal range for serum PTH level was 1.2–5.7 pmol/L.

### Statistics

For comparison of dichotomous variables between the two groups, χ^2^ test and Fisher’s exact test were used. The Mann–Whitney U test was used for comparison of continuous variables. Any preoperative and postoperative day 0 biochemical variables that were significant in the univariate analysis were entered into multivariate analysis to determine independent factors. To improve clinical utility of significant continuous variables, Youden’s index was used to calculate the best cutoff value for predicting hypocalcemia [15]. The area under a receiver characteristic (ROC) curve (AUC) was used to measure the relative predictability of these variables or criteria. AUC values close to 1.00 meant better predictability and those close to 0.500 meant poorer predictability. All statistical analyses were conducted using SPSS ver. 18.0 (SPSS, Inc., Chicago, IL, USA). *p* < 0.05 was considered statistically significant.

## Results

The median age of our cohort of 117 patients was 50.0 (range = 16.1–84.2) and the male:female ratio was 1:8.8. One-hundred nine patients (93.2 %) underwent a total thyroidectomy while 8 (6.8 %) had a completion total thyroidectomy. The median hospital stay was 2 (range = 2–5) days. Of the 17 patients who required supplements on discharge, 11 required calcium tablets alone while 6 required both calcium and calcitriol supplements. On follow-up, no patient experienced unexpected hypocalcemic symptoms or required readmission. After a median follow-up of 6.2 (range = 3.6–9.4) months, 15 of 17 (88.2 %) patients were successfully taken off supplements while 2 patients continued to take calcium and calcitriol. Since these two (1.7 %) patients took supplements for >6 months, they were regarded as having permanent hypoparathyroidism.

Table 1 compares patient baseline characteristics, surgical indication, and operative findings between group I and group II. Median age and sex ratio were similar between the two groups. Group II had a higher proportion of Graves’/toxic multinodular goiter (MNG) (29.4 vs. 10.0 %, *p* = 0.043) and a lower proportion of benign thyroid pathology than group I (52.9 vs. 80.0 %, *p* = 0.028). The overall rates of postoperative hypocalcemia in patients with malignancy, Graves’/toxic MNG, and benign pathology were 30.0, 33.3, and 10.1 %, respectively. The incidence of concomitant autoimmune thyroiditis, operative blood loss, and the weight of the excised thyroid gland were similar between the two groups, but the duration of the operation was significantly longer in group II, perhaps reflecting the difficulty of the procedure. Group II had a significantly greater number of parathyroid glands identified (*p* = 0.017) and a greater number of parathyroid glands autotransplanted (*p* = 0.019) than group I.

**Table 1:** Comparison of patient baseline characteristics, surgical indication, and operative findings between those with normocalcemia (i.e., no signs of hypocalcemia and adjusted calcium ≥1.90 mmol/L) (group I) and those with hypocalcemia (i.e., with either hypocalcemic symptoms and/or adjusted calcium <1.90 mmol/L) (group II) after bilateral thyroid resection

Variable	Group I (*n* = 100)	Group II (*n* = 17)	*p* value
Age at operation (years)	51.2 (17.9–84.3)	45.0 (15.8–68.4)	0.124
Gender (male:female)	11:89	1:16	1.000
Surgical indication/final pathology			
Malignancy	10 (10.0)	3 (17.6)	0.400
Graves’ disease/toxic MNG	10 (10.0)	5 (29.4)	**0.043**
Benign pathology	80 (80.0)	9 (52.9)	**0.028**
Concomitant autoimmune thyroiditis	10 (10.0)	2 (11.8)	0.686
Duration of operation (min)	56.5 (41–175)	72.5 (39–155)	**0.012**
Blood loss (mL)	10 (10–200)	10 (10–30)	0.080
Weight of excised gland (g)	30 (7–225)	38 (18–73)	0.083
No. of parathyroid glands identified			**0.017**
None	14 (14.0)	0 (0.0)	
1	6 (6.0)	1 (5.9)	
2	27 (27.0)	0 (0.0)	
3	2 (2.0)	3 (17.6)	
4	51 (51.0)	13 (76.5)	
No. of parathyroid glands auto-transplanted			**0.019**
None	84 (84.0)	10 (58.8)	
1	13 (13.0)	6 (35.3)	
2	3 (3.0)	1 (5.9)	

Bold values indicate statistically significant at *p*-values <0.05

*MNG* multinodular goiter

Table 2 compares preoperative thyroid function, thyroid auto-antibodies, and the perioperative biochemical profile between groups I and II. Preoperative TSH level and antithyroid auto-antibodies titer were similar between the two groups. Relative to group I, group II had significantly lower preoperative adjusted Ca (2.22 vs. 2.29 mmol/L, *p* < 0.001), postoperative 1-h adjusted Ca (2.10 vs. 2.18 mmol/L, *p* < 0.001), and postoperative 16-h adjusted Ca (1.94 vs. 2.19 mmol/L, *p* < 0.001). In terms of PTH levels, group II had significantly lower PTH-SC (3.3 vs. 3.7, *p* < 0.001) and PTH-D1 (0.1 vs. 0.6, *p* < 0.001) than group I. Overall, the PTH-SC level was significantly lower than PTH-D1 (*p* < 0.001).

**Table 2:** Comparison of preoperative thyroid function, thyroid auto-antibodies, and perioperative biochemical profile between those with normocalcemia (i.e., no signs of hypocalcemia and adjusted calcium ≥1.90 mmol/L) (group I) and those with hypocalcemia (i.e., with either hypocalcemic symptoms and/or adjusted calcium <1.90 mmol/L) (group II) after bilateral thyroid resection

Variable	Group I (*n* = 100)	Group II (*n* = 17)	*p*
Preoperative TSH level (mIU/L)	0.81 (0.03–2.80)	0.87 (0.04–2.20)	0.781
Antithyroglobulin antibody (titer)			0.130
<100	70 (70.0)	12 (70.6)	
100–400	15 (15.0)	0	
400	5 (5.0)	5 (29.4)	
Antimicrosomal antibody (titer)			0.130
<100	70 (70.0)	12 (70.6)	
100–400	15 (15.0)	0 (0.0)	
>400	5 (5.0)	5 (29.4)	
Preoperative adjusted calcium (mmol/L)	2.29 (2.14–2.50)	2.22 (2.16–2.33)	**<0.001**
Postoperative 1-h adjusted serum calcium (mmol/L)	2.18 (1.97–2.37)	2.10 (1.99–2.24)	**<0.001**
Postoperative 24-h adjusted calcium (mmol/L)	2.19 (2.01–2.33)	1.94 (1.78–2.07)	**<0.001**
Calcium drop from preoperative to postoperative 1 h (%)	4.74 (−1.82–16.00)	6.94 (1.36–10.76)	0.067
Calcium drop from postoperative 1–24 h (%)	−0.46 (−9.50–7.11)	8.92 (−2.99–14.48)	**<0.001**
Postoperative PTH at skin closure (PTH-SC) (pmol/L)	3.7 (0.3–16.3)	3.3 (0.1–18.0)	**<0.001**
Postoperative PTH on postoperative day 1 (PTH-D1) (pmol/L)	0.6 (0.2–4.6)	0.1 (0.1–4.9)	**<0.001**

Bold values indicate statistically significant at *p*-values <0.05

*TSH* thyrotropin, *PTH* intact parathyroid hormone level

Table 3 gives a multivariable analysis of clinicopathological risk factors and postoperative day 0 biochemical indicators for postoperative hypocalcemia requiring calcium ± calcitriol supplementation after surgery. For postoperative day 0 biochemical indicators, only postoperative 1-h adjusted Ca and PTH-SC were considered. After adjusting for other significant factors, low preoperative adjusted Ca [ß coefficient = −17.7, odds ratio = 0.001 (95 % CI: 0.001–0.502), *p* = 0.041] and low PTH-SC [ß coefficient = 2.063, odds ratio = 7.872 (95 % CI: 2.239–27.669), *p* = 0.001] were the two independent variables associated with hypocalcemia.

**Table 3:** Multivariable analysis of clinicopathological risk factors and postoperative day 0 biochemical indicators for postoperative clinically relevant hypocalcemia (i.e., either with hypocalcemic symptoms and/or adjusted calcium <1.90 mmol/L) requiring calcium and/or vitamin D supplementation after surgery

Covariates	ß-coefficient	Odds ratio (95 % CI)	*p*
Surgical indication			
Malignancy		1	0.122
Graves’ disease/toxic MNG	−0.476	0.622 (0.011–36.590)	0.819
Benign	2.482	11.959 (0.273–523.99)	0.198
Duration of operation (min)	0.001	1.001 (0.959–1.044)	0.981
No. of parathyroid glands seen			
None		1	0.813
1–2	1.182	3.260 (0.031–345.683)	0.619
3–4	1.035	2.815 (0.102–78.018)	0.541
Parathyroid autotransplantation	0.004	1.004 (0.096–10.484)	0.817
Preoperative adjusted calcium (mmol/L)	−17.672	0.001 (0.001–0.502)	**0.041**
Postoperative 1-h adjusted serum calcium (mmol/L)	1.448	4.254 (0.001–926591)	0.817
Postoperative PTH at skin closure (PTH-SC) (pmol/L)	2.063	7.872 (2.239–27.669)	**0.001**

Bold values indicate statistically significant at *p*-values <0.05

*MNG* multinodular goiter, *PTH* intact parathyroid hormone, *CI* confidence interval

**Fig. 1:**
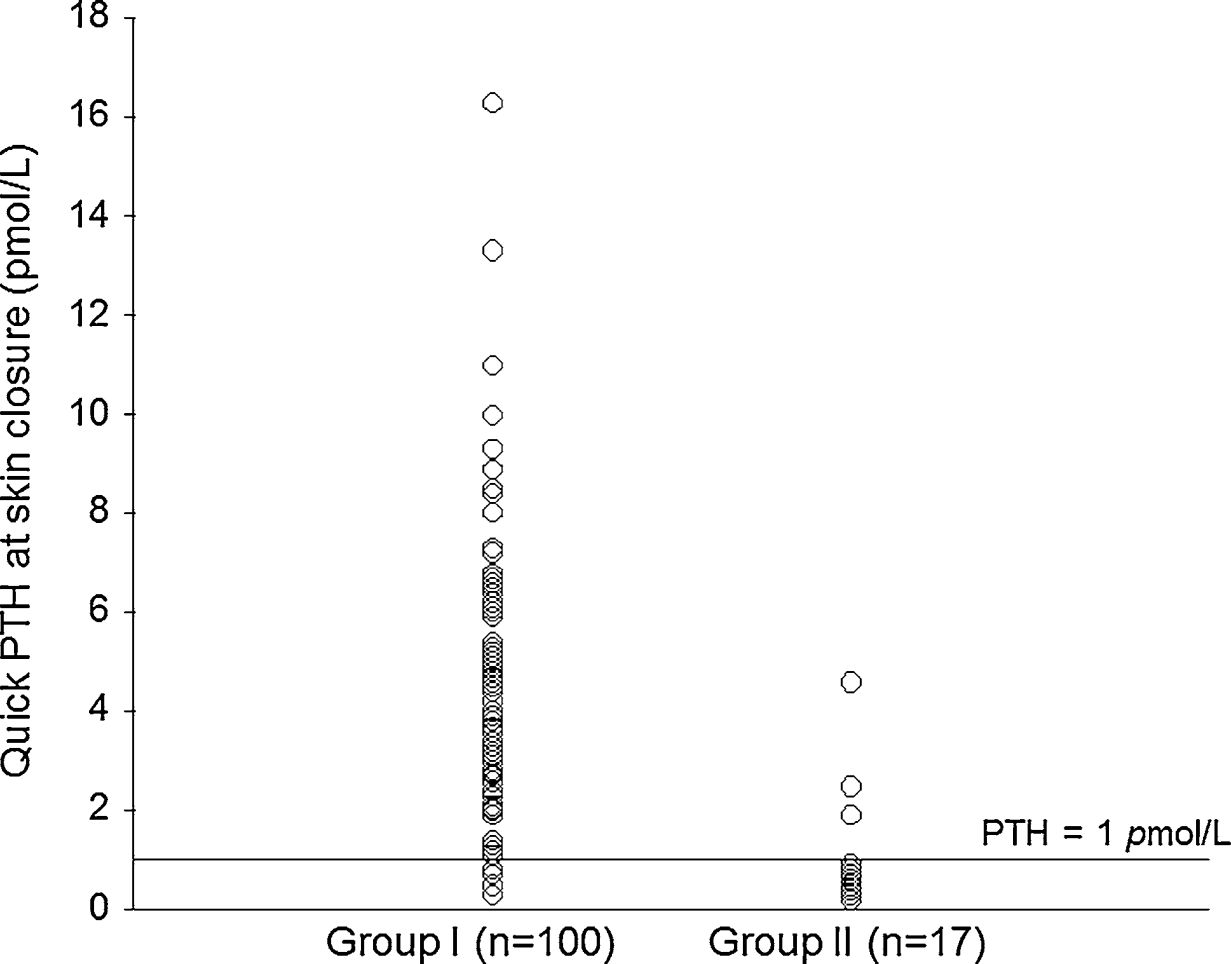
The quick parathyroid hormone levels at the time of skin closure (PTH-SC) in those with normocalcemia (i.e., no signs of hypocalcemia and adjusted calcium ≥1.90 mmol/L) (group I) and those with hypocalcemia (i.e., with either hypocalcemic symptoms and/or adjusted calcium <1.90 mmol/L) (group II) after bilateral thyroid resection

Figure 1 shows the PTH-SC for groups I and II after surgery.

Table 4 compares test sensitivity, specificity, and predictability as measured by area under the receiver operating characteristic curve (AUC) between serum Ca slopes, preoperative adjusted Ca, PTH-SC, PTH-D1, and a combined preoperative Ca and PTH-SC score. In predicting hypocalcemia, the best cutoff values for PTH-SC and PTH-D1 were ≤1.0 pmol/L (Youden’s index = 0.774; sensitivity 82.4 % and specificity 95.0 %) and ≤0.6 pmol/L (Youden’s index = 0.695; sensitivity 76.5 % and specificity 93.0 %), respectively. Interestingly, the test sensitivity, specificity, and AUC were exactly identical between PTH-SC ≤1 or >1 pmol/L and combined score ≤−1.4/> −1.4, implying that adding preoperative adjusted Ca to PTH-SC did not improve the sensitivity, specificity, and predictability.

**Table 4:** Comparison of test sensitivity, specificity, and predictability as measured by area under the receiver operating characteristic curve (AUC) between serum calcium slopes, preoperative adjusted calcium, parathyroid hormone (PTH) at skin closure (PTH-SC), PTH on postoperative D1 (PTH-D1), and combined preoperative adjusted calcium and PTH-SC score

	Best cutoff value^b^	Test sensitivity^c^ (%)	Test specificity^d^ (%)	AUC (95 % CI)
Calcium drop from preoperative to postoperative 1 h (%)	≤5.7	70.6	65.0	0.674 (0.537–0.812)
	>5.7			
Calcium drop from postoperative 1 h to 24 h (%)	≤2.3	94.1	80.0	0.875 (0.793–0.956)
	>2.3			
Preoperative adjusted calcium (mmol/L)	≤2.27	58.0	76.5	0.670 (0.537–0.803)
	>2.27			
Postoperative PTH-SC (pmol/L)	≤1.0	82.4	95.0	0.887 (0.777–0.996)
	>1.0			
Postoperative PTH-D1(pmol/L)	≤0.6	76.5	93.0	0.847 (0.725–0.970)
	>0.6			
Combined preoperative adjusted calcium and PTH-SC score^a^	≤−1.4	82.4	95.0	0.887 (0.777–0.996)
	>−1.4			

^a^Calculated from logistic regression. Combined score = 2.598–2.717 (Ca score) −5.301 (PTH score). For preoperative adjusted calcium ≤2.27 mmol/L, Ca score was 1 and for PTH-SC ≤1.0 pmol/L, the PTH score was 1. For other values, the score would be 0

^b^Determined by the receiver operating characteristic curve and Youden’s index

^c^Sensitivity = true positives/(true positives + false negatives)

^d^Specificity = true negatives/(true negatives + false positives)

Table 5 compares PTH-SC ≤1 or >1 pmol/L between groups I and II. Of the 17 patients in group II, 14 had PTH-SC ≤1 pmol/L and 3 had PTH-SC >1 pmol/L. The three patients with PTH-SC >1 pmol/L (1.9, 2.5, and 4.6 pmol/L) required oral calcium supplements (1,000, 1,000, and 500 mg daily, respectively) alone, whereas 6 of the 14 patients with PTH-SC ≤1 pmol/L required both calcium and calcitriol supplements to maintain normocalcemia on discharge. Of these six patients, two suffered permanent hypoparathyroidism and their PTH-SC values were 0.6 and 0.7 pmol/L, respectively. The PTH-SC level was not significantly different between those who eventually developed temporary or permanent hypoparathyroidism (*p* = 0.295).

**Table 5:** Comparison of PTH value at the time of skin closure (PTH-SC) ≤1 or >1 pmol/L between those with normocalcemia (i.e., no signs of hypocalcemia and adjusted calcium ≥1.90 mmol/L) (group I) and those with hypocalcemia (i.e., with either hypocalcemic symptoms and/or adjusted calcium <1.90 mmol/L) (group II) after bilateral thyroid resection

Variable	Group I (*n* = 100)	Group II (*n* = 17)	Total number
PTH-SC ≤1 pmol/L	5 (FP)	14 (TP)	19
PTH-SC >1 pmol/L	95 (TN)	3 (FN)	98
Total number	100	17	117

*FP* false positive, *TP* true positive, *TN* true negative, *FN* false negative

## Discussion

Transient hypoparathyroidism is a major reason for delayed hospital discharge following a total or completion total thyroidectomy because potentially life-threatening hypocalcemic symptoms may not occur until 24–48 h after surgery [3, 4]. Among the various strategies proposed to optimize the management of postoperative hypocalcemia by shortening hospital stay, PTH-directed selective supplementation is a safe and effective approach [9, 10, 13]. However, many PTH criteria have been proposed by previous authors to predict which patients are at risk of developing clinically relevant postoperative hypocalcemia [9–13]. In general, these criteria can be categorized into two main approaches, namely, focusing on the percentage drop in PTH level from the preoperative to the postoperative period or simply measuring a single postoperative PTH level some time after surgery with a cutoff value [9–13]. We chose the latter approach because there was no need for drawing blood multiple times; this reduced the clinical workload and the overall cost of PTH testing. In regard to the timing, two evidence-based reviews suggested a single PTH measurement taken any time from 10 min to several hours after surgery seemed to provide equally accurate predictive results [9, 13]. Barczynski et al. [12] compared the accuracy between PTH-SC and PTH at 4 h after surgery and found no significant difference. With the advantages of reduced pain, being logistically easier, and the PTH results coming back sooner, the accuracy and reliability of PTH-SC was evaluated.

In the first part of analysis, preoperative clinicopathological risk factors and day 0 biochemical indicators associated with hypocalcemia were evaluated in our cohort. The reason for evaluating only day 0 biochemical indicators (i.e., PTH-SC and not PTH-D1) was because we aimed to assess any potential indicators or factors that might be predictive in the setting of outpatient or ambulatory thyroidectomy (i.e., patients being discharged on the same day as surgery). Among the various significant factors such as Graves’/toxic MNG, duration of the operation, number of parathyroid glands seen, parathyroid autotransplantation, preoperative Ca, and PTH-SC, only the latter two turned out to be independent factors associated with clinically relevant hypocalcemia. To confirm whether combining these independent factors would further improve testing accuracy, preoperative Ca was entered as a covariate with PTH-SC in the logistic regression model and a formula for hypocalcemia was generated (see Table 4). However, test sensitivity, specificity, and AUC of this formula were exactly identical to those of PTH-SC alone. Therefore, in the ambulatory setting, PTH-SC ≤1/>1 pmol/L was probably the single most accurate and most reliable test in predicting clinically relevant hypocalcemia. Our analysis also compared the accuracy of PTH-SC with that of conventional serial Ca monitoring (or Ca slope) using their best cutoffs and found that PTH-SC had higher specificity and AUC than the two types of Ca slope, namely, from preoperative to postoperative 1 h and from postoperative 1 h to 24 h. Therefore, PTH-SC might not only be more accurate and reliable in predicting clinically relevant hypocalcemia than serial Ca monitoring, it might also eliminate the need for drawing blood multiple times and an overnight stay.

In our analysis, test sensitivity and specificity of PTH-SC were 82.4 and 95.0 %, respectively, and were consistent with those reported by other authors [12, 13, 16]. It was interesting to note that the overall accuracy of PTH-D1 was lower than that of PTH-SC, despite the fact that it was taken later or closer to the time of hospital discharge. It was observed that the PTH-D1 levels overall were lower than the PTH-SC levels in both groups I and II, suggesting that perhaps postoperative PTH levels fell regardless of whether the patient would eventually develop clinically relevant hypocalcemia or not. Unlike previous studies, our cutoff values were calculated from the ROC and Youden’s index in order to find the best balance between test sensitivity and specificity. Coincidentally, these cutoff values appeared similar to those reported in previous studies [9, 13, 17, 18].

However, despite these encouraging results, PTH-SC was not 100 % accurate. Most concerning was that 3/98 or 3.1 % of patients with PTH-SC >1 pmol/L still required calcium supplements on discharge. One patient had PTH-SC = 4.6 pmol/L and yet the adjusted Ca fell to 1.89 mmol/L on the following morning after surgery. One could argue that with time, this patient’s adjusted Ca might have spontaneously returned to above 1.90 pmol/L because this patient never experienced any hypocalcemic symptoms and only calcium 500 mg daily was prescribed on discharge. Nevertheless, if oral calcium and calcitriol requirements were analyzed more closely, none of the three patients with PTH-SC >1 pmol/L required both oral calcium and calcitriol on discharge, suggesting that they suffered from only mild hypocalcemia. In contrast, 6 of 14 patients with PTH-SC ≤1 pmol/L required both oral calcium and calcitriol and 2 eventually developed permanent hypoparathyroidism. Therefore, perhaps in the future, patients with PTH-SC >1 pmol/L could be discharged on the same day of surgery and be instructed to take oral calcium when hypocalcemic symptoms develop as the chance of a life-threatening episode of severe hypocalcemia would seem unlikely in this group of patients. However, the group with PTH-SC ≤1 pmol/L would need closer monitoring because even though 5/19 or 26.3 % never developed clinically relevant hypocalcemia, the other 14 required some oral calcium and/or calcitriol supplements to maintain normocalcemia. Perhaps, the safest strategy would be to keep this group of patients overnight after surgery for serial Ca monitoring. One point worth discussing is that although the quick PTH assay used in the present study may cost more than the conventional PTH assay, the former is able to produce the PTH result more quickly and, in our opinion, this helps to facilitate early hospital discharge and ambulatory surgery and reduce the overall medical cost of the operation.

## Conclusion

Preoperative Ca level and PTH-SC were the two most significant determinants of hypocalcemia after total or completion thyroidectomy. PTH-SC was more sensitive, specific, and predictive than the conventional overnight Ca monitoring and PTH-D1. Given that only 3.1 % of patients had mild hypocalcemia, it would seem a reasonable approach to discharge patients with PTH-SC >1 pmol/L on the same day of surgery as the risk of a life-threatening hypocalcemic episode is unlikely. However, we would recommend those with PTH-SC ≤1 pmol/L should have at least one overnight hospital stay for Ca monitoring.
